# Bekannte Herausforderungen und neue Möglichkeiten von Real-World-Daten

**DOI:** 10.1007/s00103-023-03826-w

**Published:** 2024-01-05

**Authors:** Katharina Schneider, Steffen Heß, Stefanie Weber

**Affiliations:** 1 Forschungsdatenzentrum Gesundheit, Bundesinstitut für Arzneimittel und Medizinprodukte, Kurt-Georg-Kiesinger-Allee 3, 53175 Bonn, Deutschland; 2 Abteilung „Kodiersysteme und Register“, Bundesinstitut für Arzneimittel und Medizinprodukte, Kurt-Georg-Kiesinger-Allee 3, 53175 Bonn, Deutschland

„Real-World-Daten“ (RWD) aus dem Versorgungskontext gewinnen in den Gesundheitswissenschaften zunehmend an Bedeutung. Trotzdem ist die Datenlandschaft in Deutschland und Europa bisher stark fragmentiert, was den Forschungszugang erschwert. Um dies zu ändern, entsteht am Bundesinstitut für Arzneimittel und Medizinprodukte (BfArM) das Forschungsdatenzentrum Gesundheit (FDZ Gesundheit), das Krankenkassenabrechnungsdaten von über 70 Mio. gesetzlich Versicherten Forschenden unter gesicherten Bedingungen zugänglich macht. Zukünftig sollen Versicherte auch ihre elektronischen Gesundheitsakten für das FDZ freigeben und weitere Daten mit den FDZ-Daten verknüpft werden können.

Die sekundäre Nutzung von Gesundheitsdaten ist jedoch nicht ohne Kontroversen, insbesondere hinsichtlich des Datenschutzes. Es gilt, den Nutzen für das Gesundheitswesen und die Verbesserung der Patientenversorgung mit dem Schutz individueller Daten vor Missbrauch abzuwägen. Datenschutz ist deshalb ein wichtiger Aspekt, der bereits im Aufbau von Infrastrukturen eine wichtige Rolle spielen muss. Für die Datenfreigabe ist das Vertrauen der Menschen in einen sicheren Umgang mit ihren Daten ein essenzieller Motivationsfaktor. Auch die Qualität und Aussagekraft der Daten sowie Interoperabilität und Verknüpfbarkeit verschiedener Quellen sind zentrale Themen. Das Gesundheitsdatennutzungsgesetz (GDNG) und die Verordnung zum European Health Data Space (EHDS) nehmen sich dieser Herausforderungen an und fördern die Nutzung von RWD durch Bereitstellung eines entsprechenden regulativen Rahmens.

Als Einstieg in das Themenheft geben *Ludwig et al.* einen Überblick über den Aufbau des Forschungsdatenzentrums Gesundheit. Dabei werden die Infrastruktur, der Datenkranz und die Zugriffsmöglichkeiten vorgestellt. Auch wird ein Ausblick auf zukünftige Weiterentwicklungen gegeben, die sich z. B. durch das GDNG und den geplanten EHDS ergeben werden.

*Krause et al.* diskutieren das Potenzial standardisierter Auswertungen großer Datenmengen am Beispiel des Projekts ReFern-01 zur Überwachung nichtübertragbarer Erkrankungen.

Ein weiterer Beitrag, von *Wicherski und Hänisch*, setzt sich mit dem Thema „Real-World-Daten“ (RWD) in der Arzneimittelzulassung und -überwachung auseinander. Als Ergänzung zu randomisierten klinischen Studien kann der Zulassungsprozess neuer Arzneimittel durch Einbeziehung von RWD insbesondere beschleunigt werden. Entsprechende Projekte im europäischen Raum werden im Beitrag mit betrachtet.

Ein Thema, das immer mehr an Relevanz gewinnt, ist der Einfluss des Klimas auf unsere Gesundheit. Auch beim Weltklimagipfel rückt das Thema in den Vordergrund. Im Beitrag von *Günster und Schmuker* wird aufgezeigt, dass gerade für dieses Thema RWD entscheidende Erkenntnisse liefern können. Durch die Verlinkung von RWD mit anderen Daten, wie z. B. regionalen Wetterdaten, kann die Analyse der Gesundheitsdaten helfen, Prävention, aber auch zeitnahe Reaktion auf gesundheitliche Risiken zu verbessern.

Wie bereits eingangs erwähnt, stellt der Datenschutz einen zentralen Aspekt der Ausgestaltung von RWD-Nutzung dar. In den drei folgenden Artikeln wird dieser Aspekt deshalb aus verschiedenen Blickwinkeln betrachtet und auch die Risiken sowie Möglichkeiten der Risikominimierung werden aufgezeigt. Im Beitrag von *Drechsler und Pauly* wird das Reidentifikationspotenzial von Einzelpersonen auch aus pseudonymisierten Daten betrachtet und es werden Ansätze zur Vermeidung der Reidentifikation aufgezeigt, unter Berücksichtigung des „Five Safes“-Konzepts, das im Artikel erläutert wird. Im Artikel von *Prasser et al.* wird dies konkret am Aufbau des FDZ Gesundheit erläutert und das Potenzial von synthetischen Daten erklärt. Ein hierzu durchgeführtes Projekt hat wertvolle Erkenntnisse geliefert, die im FDZ Berücksichtigung finden. Im dritten Beitrag zu diesem Themenkomplex werden dann von *Specht-Riemenschneider und Heineking* am Beispiel von MRT-Gehirnscans der rechtliche Rahmen und dessen Komplexität beleuchtet und das Potenzial von Datentreuhandstellen hervorgehoben.

Abschließend wird in diesem Themenheft noch der Blick über den Tellerrand gerichtet. Zum einen wird im Beitrag von *Arzideh et al.* die grenzübergreifende Zusammenarbeit im Aufbau von Forschungsdatenzentren betrachtet. Am Beispiel der deutsch-französischen Kooperation werden das Potenzial für Synergien und die Unterschiede aufgrund von verschiedenen nationalen Gegebenheiten herausgearbeitet. Zum anderen wird im Beitrag von *Weber *der Blick auf die Primärdatenerhebung und deren Einfluss auf die Aussagekraft von Sekundärdaten geworfen und für den holistischen Blick auf Datenerhebung und -auswertung geworben.

Dieses Themenheft hebt die Komplexität, Herausforderungen und den enormen Nutzen von RWD in der Gesundheitsforschung hervor. Angesichts der fortschreitenden Digitalisierung im Gesundheitswesen und der sich entwickelnden Gesetzgebung bietet dieses Forschungsfeld immense Möglichkeiten zur Verbesserung der Gesundheitsversorgung. Natürlich hängt hierbei der Erfolg maßgeblich von der gemeinsamen (internationalen) Anstrengung und dem Wissensaustausch zwischen verschiedenen Akteuren ab. Und solange allen die damit einhergehenden Risiken bekannt sind und diese bereits in der Konzeption großer Datenzentren beachtet werden, ist das Potenzial der Analyse von RWD auch für die datenspendenden Personen erheblich.

